# Biological evaluation of synthesized allicin and its transformation products obtained by microwaves in methanol: antioxidant activity and effect on cell growth

**DOI:** 10.1080/13102818.2014.994267

**Published:** 2015-01-08

**Authors:** Dušica P. Ilić, Sanja Stojanović, Stevo Najman, Vesna D. Nikolić, Ljiljana P. Stanojević, Ana Tačić, Ljubiša B. Nikolić

**Affiliations:** ^a^ Department of Chemistry and Chemical Technology, Faculty of Technology, University of Niš, Leskovac, Serbia; ^b^ Department for Cell and Tissue Engineering, Institute of Biology and Human Genetics, Faculty of Medicine, University of Niš, Niš, Serbia

**Keywords:** allicin, transformation products, antioxidant activity, HeLa cells, proliferation, viability

## Abstract

Allicin is the most biologically active substance present in garlic. It can be synthesized or obtained by extraction of fresh garlic. Transformation products of allicin are also biologically active. The aim of this study was to examine the antioxidant activity of synthesized allicin and its transformation products obtained using microwaves in methanol at 55 °C as well as their effect on HeLa cells growth. The antioxidant activity was determined by DPPH (2,2-diphenyl-1-picrylhydrazyl radical) test. The effect on HeLa cells growth was determined by MTT (3-(4,5-dimethylthiazole-2-yl)-2,5-diphenyl-2H-tetrazolium bromide) test. For MTT test, allicin and its transformation products were dispersed in carmellose sodium solution and examined in concentrations ranging from 0.3 μg/mL to 3 mg/mL. Allicin showed stronger antioxidant activity than the transformation products. A maximum degree of neutralization of DPPH radicals, about 90%, was reached when the concentration of allicin was 2 mg/mL, with an EC_50_ (concentration of sample which is required for reduction of the initial concentration DPPH radicals to 50%) value of 0.37 mg/mL. In our study, allicin and its transformation products were not cytotoxic to HeLa cells under the examined conditions. The highest concentration of allicin and its transformation products had a slight antiproliferative effect, with a more pronounced effect of allicin, which reflected on the morphology of HeLa cells. The examined substances are safe to use on epithelial cells at concentrations up to 3 mg/mL when applied in carmellose sodium solution. Using carmellose sodium as a dispersing agent could be recommended as a good approach for testing liposoluble substances in liquid cell cultures.

## Introduction

Allicin (3-prop-2-enylsulfinylsulfanylprop-1-ene) is a thioester of sulphonic acid, or allyl-thiosulfinate.[1] Allicin is the most important and the most active substance present in garlic. It can be synthesized or obtained by extraction of fresh garlic.[2] Because of its instability, the isolation of allicin from garlic is a very complex and difficult process.[3] Detailed investigations on the mechanisms and kinetics of allicin synthesis have been reported by Nikolić et al.[4] Allicin exhibits strong antioxidant activity [5,6] and shows cytostatic activity *in vitro*.[7] In our previous studies, the kinetic parameters of allicin transformation under the influence of ultrasonic and microwaves, along with the conventional methods at different temperatures in different polarity solvents, were determined.[1] We identified the products of allicin transformation, using preparative high-pressure liquid chromatography (HPLC).[1,8] Based on these studies, we concluded that the best way for complete allicin transformation was under the effect of microwaves in methanol. It is shown that ajoenes (*E*, *Z*-ajoen), the most common transformation products of allicin, have antioxidant activity,[9] antiproliferative activity and induce apoptosis.[10]

In this study, we examined the antioxidant activity of synthesized allicin and its transformation products obtained under the effect of microwaves in methanol as well as their effect on cell growth *in vitro*. To the best of our knowledge, there is no data about this in the available literature. The usual way of preparing the liposoluble substances, such as the studied substances, and extracts for *in vitro* testing is by pre-dissolving them in organic solvents, such as dimethyl sulfoxide (DMSO), which is toxic to cells in amounts above 0.5%.[11–13] This can be a limiting factor if higher concentrations of liposoluble substances or extracts need to be tested. Since methylcellulose is normally used for cultivation of different types of cells in semi-solid cultures and also as a gelling agent for topical preparations, we examined these substances, using carmellose sodium as a dispersing agent. This is not the common way of preparing liposoluble substances for *in vitro* examination of cytological effects in liquid cultures. Therefore, this study is also particularly important as a new approach in the examination of these liposoluble substances.

## Materials and methods

### Synthesis of allicin

Allicin was synthesized according to the procedure previously described by us.[4]

### Transformation of allicin in methanol

The transformation of allicin in methanol was performed by using microwaves in a ‘Discover’ focus microwave reactor (CEM Corporation, Matthews, NC, USA), at a frequency of 2.45 GHz, with a power of 150 W, at 55 °C.[1,8] The transformation products were isolated and their structure was characterized by HPLC, proton nuclear magnetic resonance, carbon nuclear magnetic resonance, Fourier transform infrared spectroscopy, mass spectrometry and ultraviolet (UV) spectrophotometry methods, according to the procedure described in details in our previous studies.[1,8]

### DPPH test

DPPH (2,2-diphenyl-1-picrylhydrazyl radical) test was used to determine the antioxidant activity of the synthesized allicin. The test is based on the ability of DPPH to react with molecules which show antioxidant activity, whereby a stable hydrogenated molecule DPPH-H is formed. Since the result is a colour reaction, the test is based on measuring the absorbance of samples on an UV/visible spectrophotometer in an appropriate solvent (methanol). The solutions of different concentrations (0.25 to 10 mg/mL for allicin and 2.5 to 25 mg/mL for its transformation products) were prepared from the basic allicin solution (12.5 mg/mL) and transformation products (25 mg/mL) and their absorbance was determined at 517 nm. Methanolic solution of DPPH radical (1 mL) with a concentration of 3 × 10^−4^ mol/L was added to the solutions of allicin and transformation products (2.5 mL) of various concentrations (0.25, 0.5, 0.75, 1.0, 2.0, 3.0, 5.0 and 10 mg/mL of allicin and 2.5, 5.0, 8.0, 10.0, 14.0, 18.0, 20.0 and 25.0 of transformation products). The samples were then incubated for 20 min in darkness at room temperature and the absorbance was measured at 517 nm. The absorbance of the control solution (1 mL of pure methanolic DPPH radical, concentration of 3 × 10^−4^ mol/L, in which 2.5 mL of methanol was added) was determined under the same conditions. Methanol was used as a background. The free radicals’ neutralization capacity was determined according to the following formula:(1) 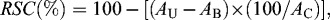
where *RSC* (%) is DPPH radical scavenging capacity, *A*_U_ is the absorbance of a sample (methanolic solution of allicin or transformation products plus DPPH radical solution) at 517 nm; *A*_B_ is the absorbance of the blank sample (methanolic solution of allicin or transformation products without addition of DPPH radical solution) at 517 nm and *A*_C_ is the control absorbance (control solution of a pure methanolic solution of DPPH).[14] The EC_50_ value is the concentration of sample (allicin or transformation products) which is required for 50% reduction of the initial DPPH concentration.

### Chemicals and reagents for cell culture

Hank's Balanced Salt Solution (HBSS), Dulbecco's Modified Eagle Medium (DMEM), stable glutamine, antibiotic–antimycotic solution, Fetal Bovine Serum (FBS) and Trypsin–EDTA (Trypsin–ethylenediaminetetraacetic acid) were purchased from PAA Laboratories (Austria), 3-(4,5-dimethylthiazole-2-yl)-2,5-diphenyl-2H-tetrazolium bromide (MTT) was purchased from Carl Roth (Germany) and 2-propanol was purchased from Fisher Chemical (USA).

### Preparing substances for testing

The tested substances were dispersed in 1% carmellose sodium solution, prepared in HBSS and the obtained solutions were diluted 1:1 in DMEM. From these stock solutions, further dilutions were made in DMEM to the final concentrations.

### Cell culture

HeLa cells were cultured in DMEM containing 10% FBS, 2 mmol/L stable glutamine and antibiotic–antimycotic solution at 37 °C in humidified atmosphere containing 5% CO_2_. The medium was replaced every 2–3 days. After reaching the appropriate confluence, the cells were detached using Trypsin–EDTA solution and centrifuged at 4 °C for 10 min at 1000 r/min. Cells were washed, counted using trypan blue dye and adjusted to an appropriate cell density.

### Viability and proliferation assays

In the viability assay, 1 × 10^5^ cells/well were seeded in 96-well plates (Sarstedt, Germany) and in the proliferation assay, 2 × 10^4^ cells/well. After 24 h of cultivation at 37 °C in an atmosphere saturated with humidity, containing 5% CO_2_, the tested substances were added in the following effective concentrations: 0.3 μg/mL, 3 μg/mL, 0.03 mg/mL, 0.3 mg/mL and 3 mg/mL. As a control we used cells that were incubated in complete DMEM without the tested substances. We have previously shown in experiments in our laboratory that carmellose sodium, in concentrations used for dispersing, does not affect the viability and proliferation of HeLa cells (unpublished data). Cells were incubated with the substances for 24 h in the viability assay and for 72 h in the proliferation assay. After the incubation period ended, cells were analysed under an inverted light microscope (AxioObserver.Z1, Carl Zeiss, Germany) and photographed with the software AxioVision version 4.6 at 100× magnification. After that the MTT test was performed in both assays.

### MTT test

MTT test is based on the reduction of yellow tetrazolium salt MTT by mitochondrial dehydrogenases of viable cells and the formation of purple formazan, which is insoluble in water. At the end of the incubation period in both assays, cells were washed with phosphate-buffered saline and then 100 μL of MTT solution was added to each well at a final concentration of 1 mg/mL. Cells were incubated in MTT solution for the next 3 h at 37 °C. Formazan crystals were dissolved with 100 μL of 2-propanol and the absorbance values were measured on Multiskan Ascent plate reader (Thermo Labsystems) at a wavelength of 540 nm.

The results are presented as percentage of cell viability or proliferation calculated using the following formula:(2) 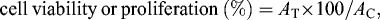
where *A*_T_ is the absorbance value of the treated culture and *A*_C_ is the absorbance value of the control culture.

### Statistical analysis

Statistical analysis was performed using the absorbance values from at least two experiments (*n* = 3 to *n* = 6 for each experiment) and included standard deviation, coefficient of variation and student's *t*-test. We considered *p* < 0.05 as significant.

## Results and discussion

### DPPH test

**Figure 1.**
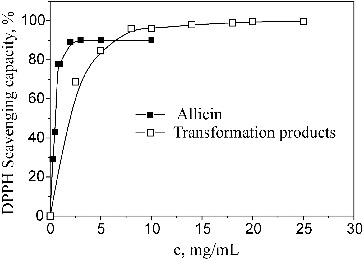
Antioxidant activity of allicin and its transformation products.

The results for the antioxidant activity of allicin and its transformation products obtained under the influence of microwaves in methanol at 55 °C are shown in Figure 1.

Allicin showed stronger antioxidant activity than its transformation products. A maximum degree of neutralization of DPPH radicals, about 90%, was reached when the concentration of allicin was 2 mg/mL with an EC_50_ value of 0.37 mg/mL. For the transformation products, a maximum degree of neutralization of DPPH radicals, about 99.61%, was reached when the concentration was 25 mg/mL while the EC_50_ value was 1.28 mg/mL.

### MTT test

**Figure 2.**
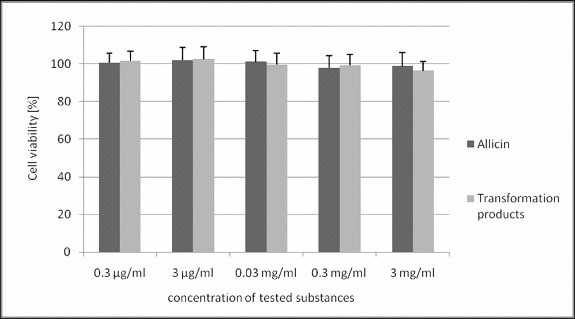
Effect of allicin and its transformation products on viability of HeLa cells.

**Figure 3.**
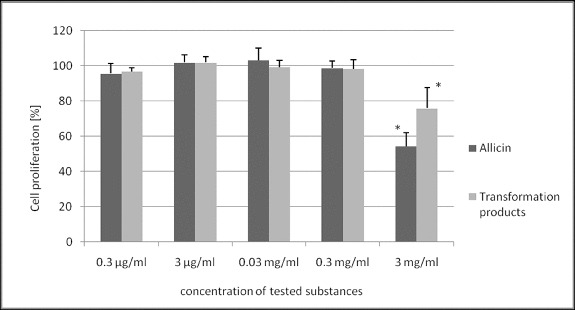
Effect of allicin and its transformation products on proliferation of HeLa cells. *p < 0.05.

**Figure 4.**
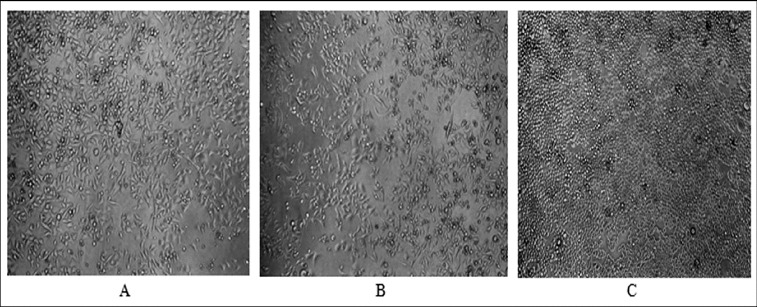
Morphology of HeLa cells after 72 h of incubation with allicin (A) and its transformation products (B) at a concentration of 3 mg/mL, as well as control culture (C); phase contrast, magnification 100×.

Allicin and its transformation products did not decrease the viability of HeLa cells (Figure 2). Only the highest concentration of allicin and its transformation products had a slight antiproliferative activity (Figure 3) with a more pronounced effect of allicin (54% and 76% of the control, *p* < 0.05). Microscopic analysis of treated cells showed no cell morphology changes in the viability assay. In the proliferation assay, however, there were morphological changes and a reduced cell count in the cultures treated with the highest concentration of the tested substances. The cells were larger, epitheloid and spindle-shaped (Figure 4).

### Final remarks

Allicin is a molecular species with much more pronounced antioxidant effect as compared to its transformation products and, as such, can find application in the preparation of pharmaceutical products. These properties of allicin demonstrated here have also been previously confirmed by other studies.[5,6] Since the reaction mixture obtained by transformation of allicin in methanol at 55 °C under the influence of microwaves contains mostly ajoene,[1,8] then the antioxidant activity of the transformation mixture could be mainly attributed to its presence [9] and the synergistic effect of ajoenes and other components present in the mixture [1,8]. It was necessary to determine the concentrations at which these substances could be safely used on epidermis. The highest concentration used in our study (3 mg/mL) was taken because it was already used in our previous experiments to examine the anti-irritating effect of allicin and its transformation products in the formulation of products for application to the skin.[15] The lowest concentration was taken because it was shown to be toxic to different cells *in vitro*, when the substances were dissolved in DMSO, in other studies.[16]

The approach employed by us for *in vitro* testing of the substances is unusual in that it uses carmellose sodium as a dispersing agent, which is commonly used as a constituent of injection preparations and topical preparations of liposoluble substances. Generally, there are reports on the cytotoxicity and antiproliferative effect of allicin dissolved in an organic solvent such as DMSO.[16,17] We have demonstrated an antiproliferative effect of relatively high concentrations of allicin and its transformation products, compared to previously published results, which may be explained at least in part by the difference in the approach used for testing of these substances.

The antiproliferative activity of allicin and its transformation products is considered to be the result of accumulation of cells in the regulatory checkpoints, G0/G1 and G2/M of the cell cycle.[18] These effects were shown in micromolar concentrations of allicin and its derivatives. In our experiment, a three-day incubation of HeLa cells with allicin and its transformation products, at the highest examined concentration, led to reduced cell growth and acted slightly antiproliferatively. One possible explanation for the antiproliferative effect at the highest concentrations may be Lawson's finding that at high concentrations allicin shows pro-oxidant effect,[19] which might cause the reaction of allicin with other reducible chemical structures in the cell. In this respect, it could be speculated that the most likely mechanism of antiproliferative activity in our study was perhaps the effect on tubulin polymerization, previously reported in other studies.[20] Allicin reacts with tubulin SH groups, which results in microtubule depolymerization in cells within minutes of its application, which makes it a potent microtubule-disrupting agent.[20] In [20], the actin stress fibres were found to be more numerous and prominent under the influence of allicin, which could very likely be the mechanism that led to the morphological changes observed in HeLa cells in our experiments.

## Conclusions

Allicin was shown to be a more potent antioxidant than its transformation products. A maximum degree of neutralization of DPPH radicals, about 90%, was reached at 2 mg/mL of allicin, with an EC_50_ value of 0.37 mg/mL, whereas its transformation products at the concentration of 25 mg/mL gave about 99.61% maximum degree of neutralization of DPPH radicals, with a much higher EC_50_ value (1.28 mg/mL). Allicin and its transformation products were not cytotoxic to HeLa cells in the examined conditions and showed a slight antiproliferative effect at a concentration of 3 mg/mL, which was more pronounced for allicin. Based on our results, these substances could be considered generally safe to use on epithelial cells, when applied in concentrations up to 3 mg/mL in carmellose sodium solution. Also, the new approach using carmellose sodium as a dispersing agent can be recommended for testing of liposoluble substances in liquid cell cultures.
